# Speech motor impairment in ALS is associated with multiregional cortical thinning beyond primary motor cortex

**DOI:** 10.3389/fneur.2024.1451177

**Published:** 2024-10-01

**Authors:** Ana Luiza Zaninotto, Meena M. Makary, Hannah P. Rowe, Marziye Eshghi, Chieh-En (Jane) Tseng, James Chan, Nicole R. Zürcher, Jacob Hooker, Austin Lewis, Mackenzie Keegan, Ryan F. Gifford, Jordan R. Green, Suma Babu

**Affiliations:** ^1^Department of Communication Sciences and Disorders, MGH Institute of Health Professions, Boston, MA, United States; ^2^Athinoula A. Martinos Center for Biomedical Imaging, Massachusetts General Hospital, Department of Radiology, Harvard Medical School, Boston, MA, United States; ^3^Systems and Biomedical Engineering Department, Cairo University, Giza, Egypt; ^4^Department of Speech, Language and Hearing Science, Boston, MA, United States; ^5^Sean M Healey & AMG Center for ALS, Department of Neurology, Boston, MA, United States

**Keywords:** amyotrophic lateral sclerosis (ALS), speech motor control, speech impairment, MRI, neurodegeneration, dysarthria, brain atrophy

## Abstract

**Introduction:**

Cortical thinning is well-documented in individuals with amyotrophic lateral sclerosis (ALS), yet its association with speech deterioration remains understudied. This study characterizes anatomical changes in the brain within the context of speech impairment patterns in individuals with ALS, providing insight into the disease's multiregional spread and biology.

**Methods:**

To evaluate patterns of cortical thickness in speakers with ALS with and without functional speech changes compared to healthy controls (HCs) using whole-brain and region of interest (ROI) analyses. Forty individuals with ALS and 22 HCs underwent a T1-weighted 3-Tesla magnetic resonance imaging (MRI). Individuals with ALS were divided into two groups based on the preserved speech [ps-ALS] (*n* = 18) or deteriorated speech [ds-ALS] (*n* = 22) as measured by the ALSFRSF-R speech subscore (=4 or <4 points, respectively). Sixteen a priori-defined and automatically segmented cortical and subcortical brain ROIs were selected based on their previously documented roles in speech production. Two cortical thickness analyses were performed: (1) group-level whole-brain surface-based analyses and (2) group-level ROI analyses. A case study of 6 ALS individuals examined the cortical thickness, and their speech was characterized using quantitative and qualitative measures.

**Results:**

Based on the group-level whole-brain surface-based analyses, the ds-ALS group demonstrated significant cortical thinning compared to HCs in the left primary motor and somatosensory cortices and the right inferior parietal lobe with its adjacent lateral occipital cortical regions. The ps-ALS group demonstrated no significant cortical thinning compared to HCs. Based on the group-level ROI analyses, the ds-ALS group demonstrated significant cortical thinning compared to HCs in bilateral middle motor cortices, right posterior dorsal premotor cortex, and left anterior cingulate cortex. The case study analysis revealed that ALS speakers with speech features characteristic of spastic dysarthria exhibited cortical thinning, while those with speech features characteristic of flaccid dysarthria did not.

**Discussion:**

Individuals with ALS have anatomical changes involving multiregional neocortical areas beyond the primary motor cortex that may manifest as subjective (i.e., clinical judgment) and objective (i.e., speaking rate) changes in speech production. Further longitudinal work in ALS is needed to better understand the link between MRI cortical thickness changes and bulbar dysfunction.

## 1 Introduction

Amyotrophic lateral sclerosis (ALS) is a neurodegenerative disease affecting both upper and lower motor neurons (UMNs and LMNs, respectively). Over the progression of the disease, impairment of the bulbar muscles often results in dysarthria, which is a neuromotor disorder of speech execution that often leads to significantly reduced speech intelligibility (i.e., the ability to be understood). Dysarthria in ALS is typically one of three types: spastic, flaccid, or mixed spastic-flaccid, all of which can have a devastating impact on quality of life (1). Flaccid dysarthria tends to be characterized by weakened, atrophied muscles, leading to slurred speech and difficulty articulating sounds, while spastic dysarthria tends to be characterized by increased muscle tone, causing strained and effortful speech due to involuntary muscle contractions (2, 3). While the different types of speech impairment secondary to ALS are known to result from UMN and LMN damage (2, 3), there is still a paucity of knowledge regarding the specific brain regions that underlie speech deterioration in ALS.

A number of studies have examined neural correlates of bulbar deficits (i.e., impairments in salivation, swallowing, and speech) in ALS and found associations with cortical thinning in the ventral motor cortex (4), premotor and supplementary motor areas (5, 6), and superior temporal gyrus (7, 8). The largest study to date relating bulbar function to brain imaging examined 192 individuals with ALS and 314 controls longitudinally (9). The authors found that cortical thinning in motor regions was detectable earlier and progressed more rapidly than clinical UMN symptoms (9). These findings suggest that cortical thinning may serve as predictive marker for clinical UMN damage and tracks disease biology in the brain better than clinical symptoms (9). Another recent study correlated speech performance, as indexed by syllable production rate task, with structural connectivity among six extramotor regions in 19 individuals with ALS (7). The authors found that degeneration of in the left speech network may relate to bulbar severity (7). While these studies provide crucial knowledge regarding the role of extramotor regions in bulbar function, they either focus on bulbar deficits more broadly, which precludes the identification of regions underlying *speech* disturbances, specifically, or they are limited in sample size and consequently investigate a limited number of brain regions.

The current study seeks to examine the relationship between cortical thinning and functional speech deterioration in a larger sample with a more comprehensive network of speech-related brain regions. For a precise yet comprehensive characterization of the neural substrates involved in speech production, we used a new segmentation cortical map: the SpeechLabel Parcellation Atlas (10). This atlas delineates several regions of interest (ROIs) and their specific roles in speech production based on speech motor impairment patterns seen across a variety of neurological disorders. While it has been increasingly used for studying speech production in speech motor-impaired populations (11–13), to date, this atlas has not yet been used to examine specific regional imaging differences as they relate to speech motor deficits in ALS.

## 2 Methods

A single-center cross-sectional 3T MRI study of forty individuals with ALS was conducted at Massachusetts General Hospital (MGH) in Boston, USA. The study was approved by the institutional review board, and all eligible participants provided written informed consent before joining the study.

### 2.1 Participants and clinical assessment

Forty individuals diagnosed with ALS and 22 sex- and age-matched healthy controls were pooled from a larger prospective multimodal neuroimaging study cohort previously published by our group (14) between May 22, 2012 and May 24, 2017. Diagnosis of possible or probable ALS was made based on the El Escorial criteria (15). Participants were excluded if they were diagnosed with other motor neuron diseases including primary lateral sclerosis, hereditary spastic paraplegia, and pure LMN phenotypes; had incomplete or missing clinical data; or did not pass MRI scan data quality check due to motion artifacts or an incomplete scan.

Standard ALS clinical assessments included participant demographics, ALS disease history, and the revised ALS Functional Rating Scale (ALSFRS-R) (16). The disease progression pre-slope, delta (Δ)ALSFRS-R was defined as [(48-ALSFRS-R score at scan)/disease duration (in months)] (16).

The ALSFRS-R speech item subscore (0–4) was used to dichotomize ALS participants into those with preserved speech (ps-ALS; ALSFRS-R speech item score = 4) and those with deteriorated speech (ds-ALS; ALSFRS-R speech item score < 4). The latter group includes individuals who reported one of the following on the ALSFRS-R speech item: detectable speech disturbance (score = 3), intelligible speech with repeating (score = 2), speech combined with nonvocal communications (score = 1), or loss of useful communication (score = 0). While all 18 participants in the ps-ALS group had preserved speech function, four participants endorsed other bulbar symptoms (1 participant rated swallow/dysphagia 2 points out of 4, and 3 participants rated drooling/sialorrhea 3 out of 4 points). All 22 participants in the ds-ALS group reported bulbar dysfunction in multiple bulbar functions (16 participants rated swallow/dysphagia 2 or 3 points out of 4, and 15 participants rated drooling/sialorrhea 0, 1, 2, or 3 out of 4). Dichotomization based on ALSRFRS-R score is a commonly used method for grouping ALS participants (17). Detailed demographic information of the participants is displayed in Table 1.

**Table 1:** Baseline demographics of the participants included in the study.

**Variables**	**HCs**	**ps-ALS**	**ds-ALS**	***p*-value**
	***N*** = **22**	***N*** = **18**	***N*** = **22**	
	**Mean** ±**SD [range]**	**Mean** ±**SD [range]**	**Mean** ±**SD [range]**	
Age at scan	50.2 ± 9.4 [33.1–65.1]	53.5 ± 12.2 [33.2–73.5]	56.9 ± 8.1 [38.9–69.6]	0.080^∧^
Age at onset	NA	51.9 ± 12.4 [33.0–73]	55 ± 7.8 [38–67]	0.100^#^
Duration of the disease (months)	NA	20.4 ± 14.2 [3.0–59.2]	28.2 ± 34.5 [1–170]	0.670^#^
Disease progression slope	NA	0.45 ± 0.41 [0.0–1.5]	0.73 ± 1.1 [0.3–5]	0.490^&^
Disease onset region (bulbar, *n*, %)	NA	0 (0%)	11 (51%)	NA
Slow vital capacity (% predicted)	NA	94.6 ± 13.5 [66–120]	77.8 ± 19.3 [22–106]	**0.003** ^**#**^
Sex (M/F)	11/12	11/5	10/11	0.170^¤^
**ALSFRS-R scale**				
ALSFRS-R at the scan time point				
Bulbar subscore (0–12)	NA	11.9 ± 0.3 [11.0–12.0]	8.4 ± 2.2 [2.0–11.0]	**< 0.010** ^#^
i. Speech item	NA	4 ± 0 [4.0]	2.2 ± 0.9 [0–3.0]	NA
ii. Swallowing item	NA	3.9 ± 0.5 [2.0–4.0]	3.1 ± 0.7 [2.0–4.0]	**0.001** ^**#**^
iii. Sialorrhea item	NA	3.8 ± 0.4 [3.0–4.0]	3 ± 1 [0–4.0]	**0.001** ^**#**^
Respiratory subscore (0–12)	NA	11.4 ± 1 [9.0–12.0]	10.9 ± 1.5 [7.0–12.0]	0.610^#^
Fine motor subscore (0–12)	NA	9.5 ± 1.3 [7.0–12.0]	9.1 ± 3.2 [0–12.0]	**0.001** ^**#**^
Gross motor subscore (0–12)	NA	7.3 ± 2.1 [5.0–12.0]	8.7 ± 3.1 [1.0–12.0]	**0.001** ^**#**^
Total ALSFRS-R score (0–48)	NA	40.2 ± 2.5 [37.0–46.0]	37 ± 6.8 [16.0–46.0]	0.200^#^

HCs, healthy controls; ps-ALS, preserved speech in amyotrophic lateral sclerosis (ALSFRS-R speech score = 4); ds-ALS, deteriorated speech in amyotrophic lateral sclerosis (ALSFRS-R speech score < 4).

^#^Wilcoxon rank-sum (Mann-Whitney) test.

^∧^One-way ANOVA test.

^&^Disease progression slope = (48 – individual ALS-FRS-R total score)/disease duration in months.

^¤^Exact Chi-square. Bold values are considered statistically significant.

Six participants with ALS were co-enrolled in a quantitative and qualitative speech assessment case study conducted by two licensed speech-language pathologists (SLPs). These participants were selected given the data we had available with speech samples. For the quantitative speech assessment, speaking rate was used given its role as one of the most widely used objective measures of bulbar impairment (18). Speaking rate was indexed by the number of words produced per minute during the Sentence Intelligibility Test (SIT), which consists of 11 randomly-generated sentences that vary from 5 to 15 words in length (19). Recent work has demonstrated an average of 198.55 (SD = 21.80) words produced per minute for typical speakers during the SIT (20). For the qualitative speech assessment, two experienced SLPs, who were blinded to imaging findings and ALSFRS-R scores, independently rated overall speech severity using a five-point equal interval scale (i.e., normal, mild, moderate, severe, and profound) and described each participant's speech based on the 38 perceptual features in the Darley, Aronson, and Brown paradigm (2, 3). The full list of features is reported in the Supplementary material.

### 2.2 MRI data acquisition and analysis

All eligible participants underwent a brain scan at the Martinos Center for Biomedical Imaging in Boston with Siemens 3 Tesla TrioTim MRI scanner equipped with an 8-channel head coil. The images were acquired using a multiple gradient echo 3-dimensional (3D) magnetization-prepared rapid acquisition (ME-MPRAGE) scan (multi-echo time TEs = [1.63, 3.49, 5.35, 7.21]; repetition time TR = 1,200; flip angle = 7 degrees; field of view FOV = 280; slice orientation = PSL; and 1 mm isotropic voxel).

Automated segmentation of cortical and subcortical structures was performed with the FreeSurfer version 6.0.26 image analysis pipeline (21). Each structural image underwent spatial and intensity normalization, skull stripping, 10-mm spatial smoothing, Talairach transformation, surface extraction, spherical registration, and cortical parcellation. The model was fitted to every vertex using Freesurfer's mri_glmfit function. Cortical thickness was then measured from the distance between the gray-white boundary and the pial surface across the entire cortical surface. The generated masks of all participants were inspected individually for accurate cortical measurement. Data were registered to FreeSurfer's standard space (fsaverage space) for conducting higher-level group analyses.

### 2.3 Speech motor ROI selection and labeling

Two approaches were used to measure cortical thinning: (1) whole-brain surface-based analyses with cluster correction, which is a powerful approach for quantifying group-level structural differences between HCs vs. each of the ALS groups (ps-ALS and ds-ALS); and (2) ROI analyses with family-wise error correction, which we used to investigate potential clinical phenotypes (as indexed by brain changes) in participants with ALS (ps-ALS and ds-ALS).

To investigate the brain regions associated with speech function in participants with ALS, 16 regions of interest (ROIs) were defined *apriori*. The selection of the ROIs was based on the involvement in speech motor control in healthy individuals and individuals with other neurodegenerative disorders as demonstrated in prior MRI and fMRI studies (11, 22). The complete Speech Label Atlas parcellate each cortical hemisphere into 64 ROIs, including a fine-grained subdivision of regions relevant to speech motor control (23). The atlas space for the extraction of the cortical thickness ROIs followed the pipeline developed by Tourville and colleagues (10).

### 2.4 Statistical analysis

#### 2.4.1 Group-level whole-brain surface-based analyses

Whole-brain surface-based analyses were performed to detect group-level differences in cortical thickness. To correct for multiple testing, we applied a cluster-wise correction with a cluster-forming threshold of 1.3 (*p* < 0.05) and a cluster-wise threshold of *p* = 0.05. A general linear model (GLM) was used for pairwise comparisons between ps-ALS vs. HC groups, and ds-ALS vs. HC groups. Age and sex were added to the model as covariates. We compared each ALS group (ps-ALS and ds-ALS) to controls separately to explore the specific effects of speech performance and cortical thickness in persons with ALS.

#### 2.4.2 Group-level region of interest analyses

Region of interest (ROI) analysis was performed using a linear regression model to detect group-level differences (ps-ALS vs. HCs, and ds-ALS vs. HC) in 16 cortical thickness ROIs. Age and sex were added to the model as covariates. To correct for multiple comparisons, we used Romano-Wolf Multiple Hypothesis Correction step-down adjusted *p*-values with 100 permutations. The uncorrected and corrected *p*-values of the ROI analyses are reported in the Supplementary material.

#### 2.4.3 Individual speech phenotype-imaging correlations

For the case study speech analyses for the six pre-identified ALS participants, a whole-brain surface-based analysis was used in a singleton approach by comparing each participant's image against HCs (*n* = 22). To correct for multiple comparisons, we applied a cluster-wise correction with a cluster-forming threshold of 1.3 (*p* < 0.05) and a cluster-wise threshold of *p* = 0.05. The cluster-forming threshold was obtained from the *z*-score sampling distribution of the largest null hypothesis cluster size, and the cluster-wise *p*-value was computed based on the number of clusters that exceeded the threshold. Age and sex were added to the model as covariates.

## 3 Results

No group differences were observed in baseline demographic characteristics. There were no significant differences between the ps-ALS and ds-ALS groups in their disease characteristics, except region of disease onset. The ds-ALS group had significantly more individuals with bulbar onset, as would be expected from the presence of speech deterioration. All participants exhibited a slow rate of progression, except for two participants in the ds-ALS group (ALSFRS-R pre-slope of >-2 points/month). The ds-ALS group had a significantly higher proportion of bulbar onset and lower bulbar ALSFRS-R subscores compared to the ps-ALS group (Table 1).

### 3.1 Group-level whole-brain surface-based analyses

**Figure 1:**
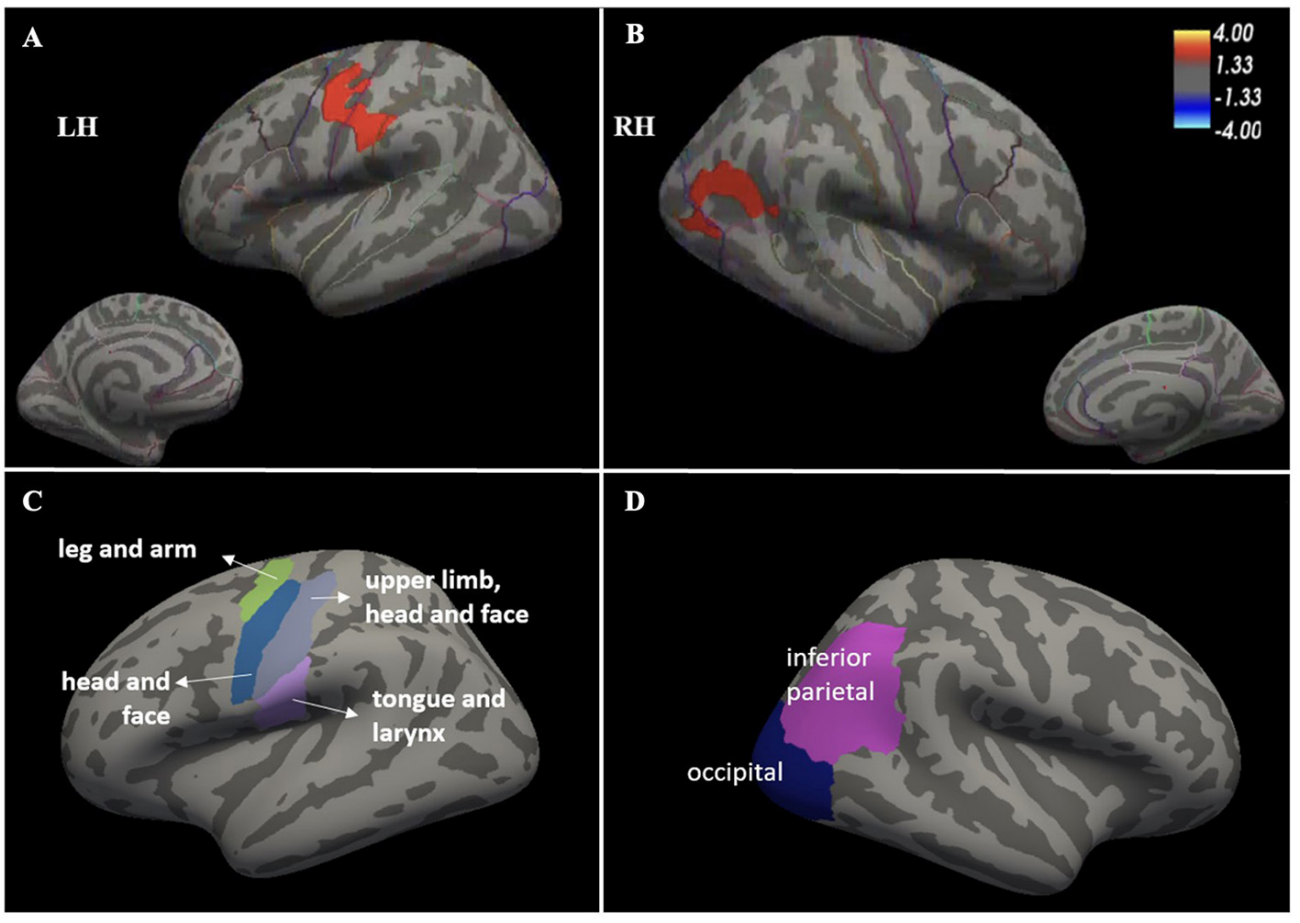
Whole-brain surface-based analysis depicting significant cortical thinning in ds-ALS group compared to HCs in left primary motor and somatosensory cortices (peak MNI coordinates *X* = −59.9, *Y* = −14.4, *Z* = 32.4) **(A)** [see anatomical subdivisions in **(C)**] and right inferior parietal lobe with its adjacent lateral occipital cortical regions (peak MNI coordinates *X* = 49.5, *Y* = −57.3, *Z* = 18.2) **(B)** [see anatomical subdivisions in Panel **(D)**]. Values indicated on the color bar are –log(*p*). ds-ALS, deteriorated speech in amyotrophic lateral sclerosis; HCs, healthy controls; RH, right hemisphere; LH, left hemisphere.

Among all brain regions analyzed on whole-brain surface-based analyses, the ds-ALS group showed significant (*p* < 0.05) cortical thinning compared to HCs, surviving multiple comparisons, in left pre-central cortex (or primary motor cortex), left post-central cortex (or primary somatosensory cortex), and right inferior parietal lobe with its adjacent lateral occipital cortical regions (Figure 1). There were no detectable differences in cortical thinning between the ps-ALS group and HCs.

### 3.2 Group-level region of interest analyses

**Figure 2:**
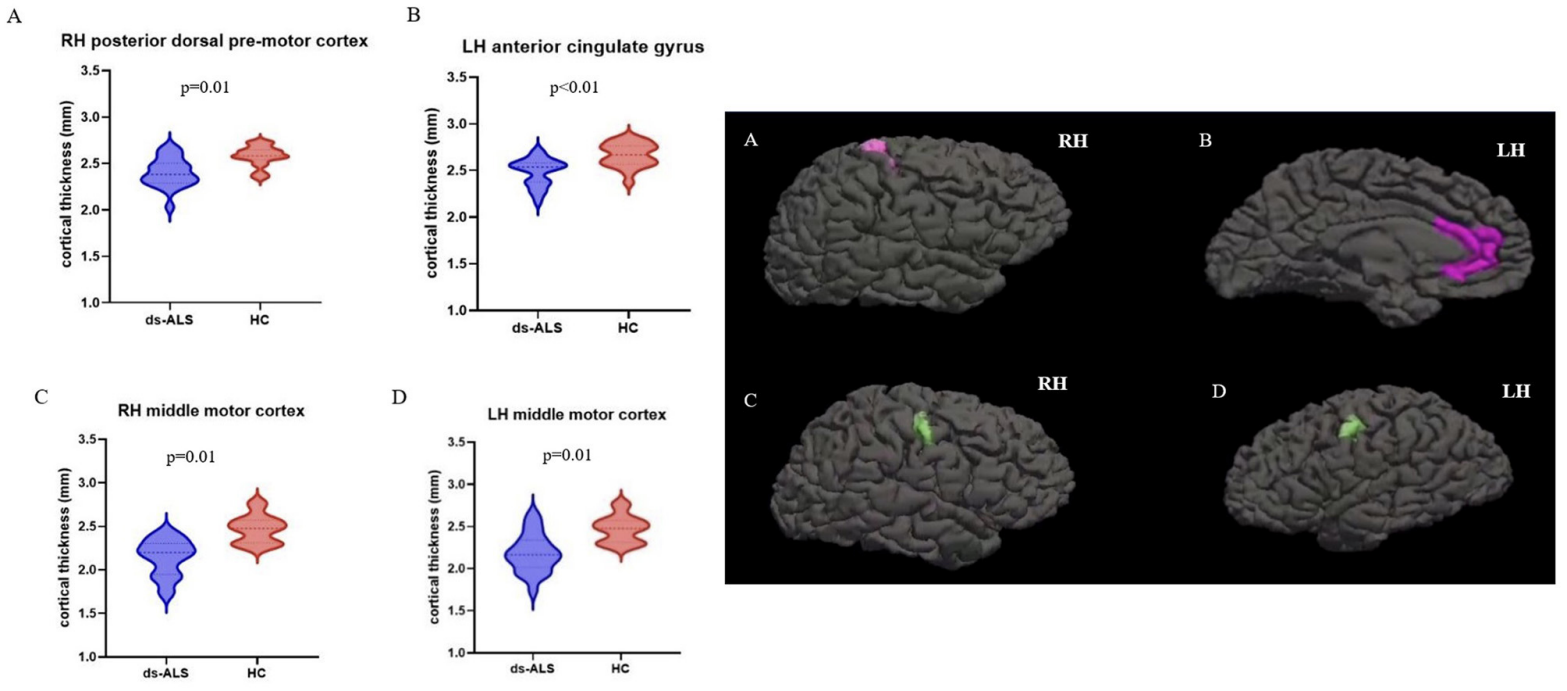
ROI analysis with anatomical maps depicting significant cortical thinning in ds-ALS group compared to HCs in right posterior dorsal premotor cortex **(A)**, left anterior cingulate gyrus **(B)**, right middle motor cortex **(C)**, and left middle motor cortex **(D)**. ROI, region of interest; ds-ALS, deteriorated speech in amyotrophic lateral sclerosis; HCs, healthy controls; RH, right hemisphere; LH, left hemisphere.

Compared to HCs, the ds-ALS group showed significant cortical thinning in right posterior dorsal premotor cortex, left anterior cingulate cortex, and right and left middle motor cortices after adjusting for multiple comparisons (Figure 2). The ps-ALS group, in contrast, showed no regional differences compared to HCs in any of the 16 ROIs after multiple comparison testing.

### 3.3 Individual speech phenotype-imaging correlations

Qualitative and quantitative speech phenotyping was performed at a matching timepoint to the scan for six ALS participants (Table 2). Four of the six participants experienced speech difficulties as reported by the ALSFRS-R as well as by the SLP-administered speech assessments (Table 2).

**Table 2:** Clinical characteristics of the ALS individual speech analyses dataset.

**ALS participant**	**Age**	**Sex**	**Place of onset**	**Disease duration (months)**	**Genetic status**	**ALSFRS-R total score (range 0–48)**	**ALSFRS-R bulbar subscore (range 0–12)**	**Disease slope**	**SVC**	**ALSFRS-R speech subscore (range 0–4)**	**Regions with significant cortical thinning**	Speech severity^*^	Perceptual speech assessment based on DAB^*^	**Speaking rate (wpm)**
#1	53	M	Bulbar	11	Unknown	47	11	0.09	86	3	None	Mild	Imprecise Articulation	196.94
#2	70	M	Limb	3	SOD1G94A	42	12	2	NA	4	None	Normal	No speech issues	181.82
#3	49	M	Limb	34	Unknown	38	11	0.3	98	3	None	Mild	Imprecise articulation, hypernasality, breathy voice	147
#4	56	F	Bulbar	11	C9orf72	37	10	1	53	3	Right SP, PCu, Cu, PR, SF, CMF	Moderate to severe	Slow rate, strained voice, imprecise articulation	103
#5	64	F	Limb	22	Unknown	34	9	0.65	81	3	Right PCu, isthmus cingulate	Severe	Slow rate, strained voice, rough voice	67.92
#6	54	M	Limb	6	SOD1 I114T	37	12	2.83	87	4	Left SMG, IP, STS	Normal	No speech issues	175.1

ALSFRS-R, Revised Amyotrophic Lateral Sclerosis Functional Rating Scale-Revised; SVC, slow vital capacity in percentage predicted; DAB, Darley, Aronson, and Brown paradigm; wpm, words per minute; F, female; M, male; NA, not available; SP, superior parietal; PCu, prenueus; Cu, cuneus; PR, precentral; SF, superior frontal; CMF, caudal middle frontal; SMG, supramarginal gyrus; IP, inferior parietal; STS, superior temporal sulcus.

^*^Completed and validated by licensed speech-language pathologists HPR and ME.

Based on the qualitative assessment conducted by two SLPs, two of the four participants with speech difficulties (#4 and #5) exhibited predominantly spastic dysarthria symptoms characterized by slow speaking rate on the SIT and strained quality voice; one (#4) also exhibited mild combined UMN-LMN features characterized by articulatory imprecision. The remaining two participants with speech difficulties (#1 and #3) exhibited predominantly flaccid dysarthria speech patterns; one (#1) presented with only articulatory imprecision, while the other (#3) presented with articulatory imprecision, hypernasality, and breathy voice, all of which are characteristic of flaccid dysarthria. Participants #2 and #6 did not exhibit speech symptoms.

**Figure 3:**
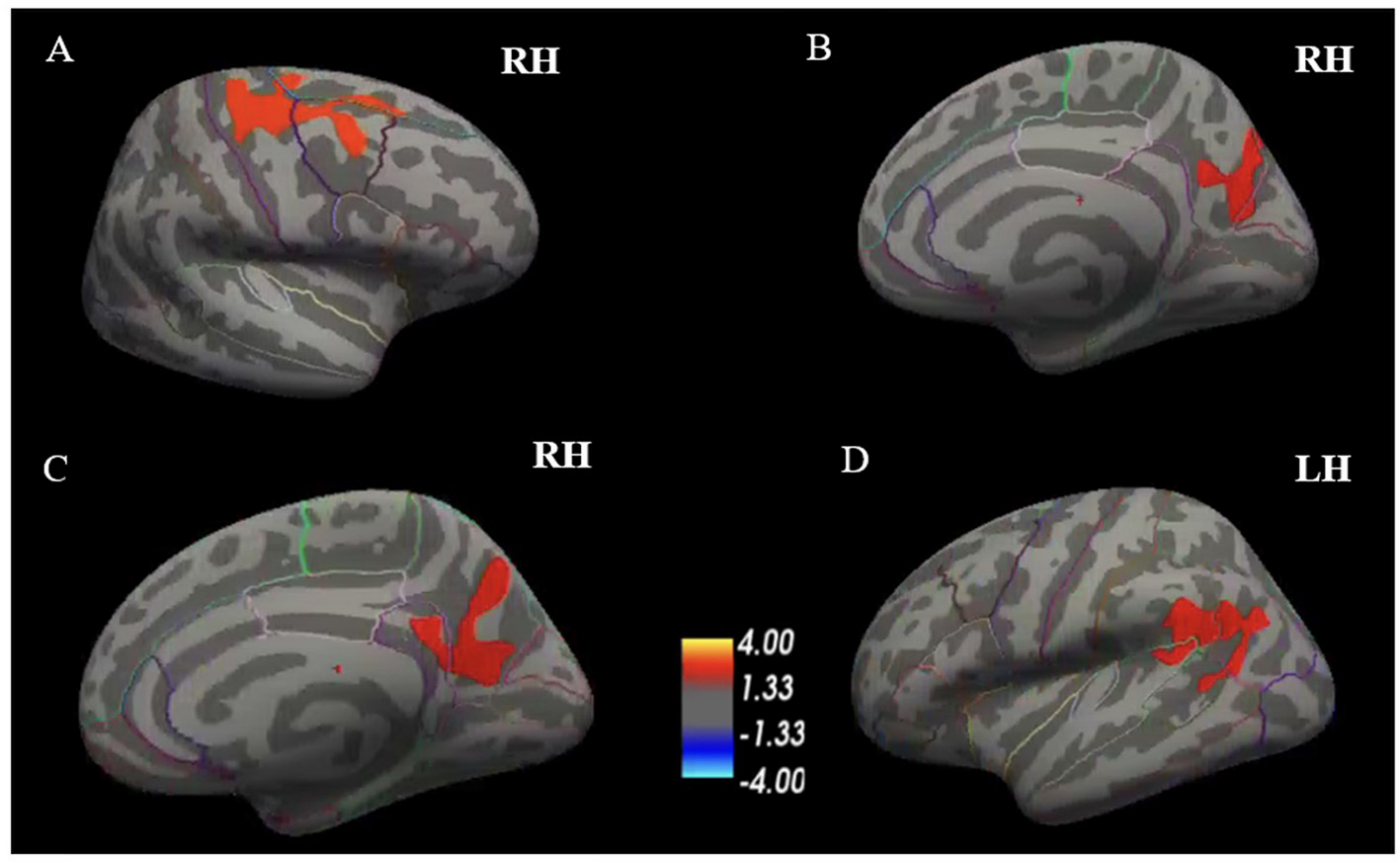
Whole-brain surface-based analysis depicting significant cortical thinning in the ALS participants included in the case study compared to HCs. Participant #4, who presented with moderate to severe speech disturbances and predominant spastic dysarthria, exhibited cortical thinning in right precentral, right superior frontal, and right caudal middle frontal regions **(A)** and in right superior parietal, precuneus, and cuneus regions **(B)**. Participant #5, who presented with severe speech disturbances and predominant spastic dysarthria, exhibited cortical thinning in right precuneus and isthmus cingulate **(C)**. Participant #6, who presented with no speech disturbances, exhibited cortical thinning in left supramarginal (inferior parietal) and superior temporal regions **(D)**. Values indicated on the color bar are –log(*p*). ALS, amyotrophic lateral sclerosis; HCs, healthy controls; RH, right hemisphere; LH, left hemisphere.

Cortical thinning was observed in two of the four participants with speech difficulties and one of the participants with no speech difficulties (Figure 3). Participant #4 (spastic-dominant) had significant cortical thinning in the right precentral, right superior frontal, and right caudal middle frontal regions, which are involved in speech planning and initiation (Figure 3A) and the right superior parietal, precuneus, and cuneus regions, which are involved in speech motor perception and rhythm regulation (Figure 3B) (54). Participant #5 (spastic-dominant) had significant cortical thinning in the right precuneus and isthmus cingulate (Figure 3C). Participant #6 (no clinical speech deficits) had significant cortical thinning in the left supramarginal (inferior parietal) and superior temporal regions. Neither participants with flaccid-dominant dysarthria (#1 and #3) nor participant #2 (no clinical speech deficits) had significant cortical thinning compared to HCs.

**Figure 4:**
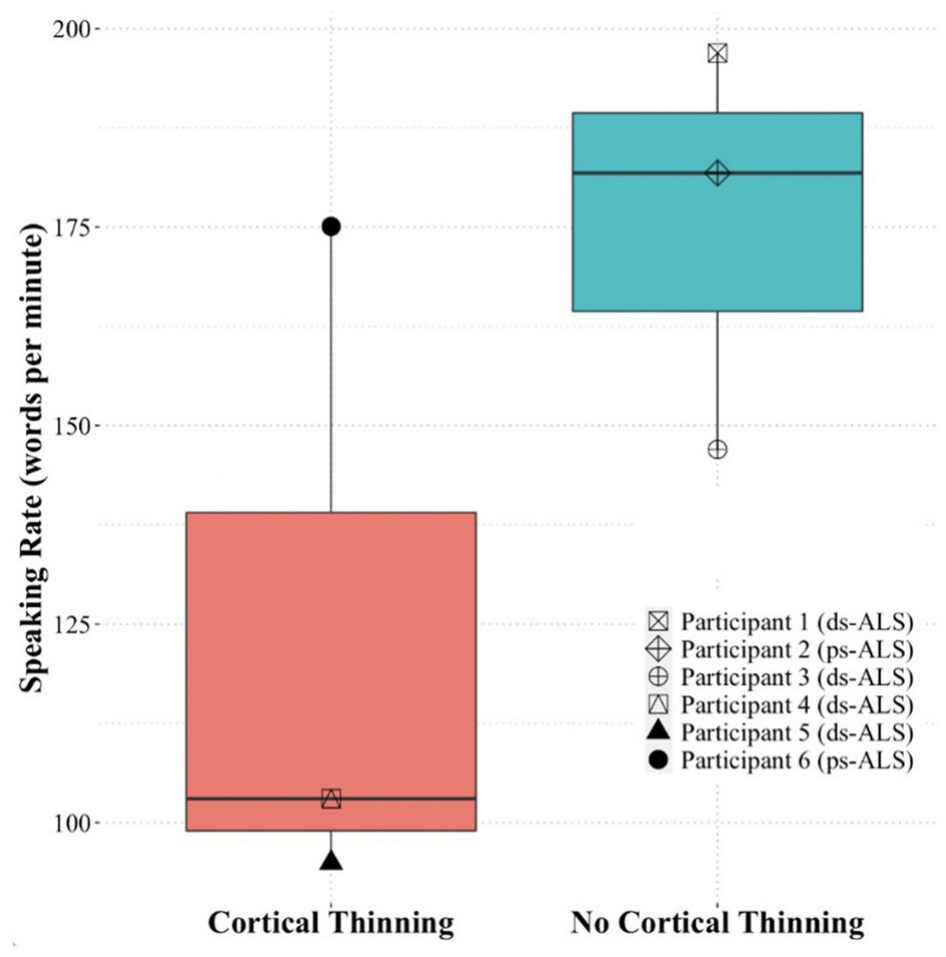
Speaking rate of the ALS participants included in the case study analysis based on presence of cortical thinning (participants #4, #5, and #6) or absence of cortical thinning (participants #1, #2, and #3). ALS, amyotrophic lateral sclerosis.

Based on the quantitative speech rate analyses, we found a large effect size difference (Cohen's *d* = 1.41) between the three ALS participants (#4, #5, and #6) with cortical thinning in speech areas and the three ALS participants (#1, #2, and #3) without cortical thinning changes in speech areas on singleton analyses against pooled HCs (Figure 4). Due to our small sample size, we did not examine statistical differences between the groups.

## 4 Discussion

Our findings provide early insights into the multifocal involvement of neocortical brain regions (beyond primary motor cortices) in speech dysfunction in individuals with ALS. Compared to HCs, the ds-ALS group exhibited cortical thinning in multiple specific neocortical regions known to be involved in speech motor production, while the ps-ALS group exhibited no changes in cortical thickness in any speech-related regions. The group-level whole-brain surface-based analyses revealed significant cortical thinning in only the ds-ALS group compared to HCs in (1) left primary motor and somatosensory cortices and (2) right inferior parietal lobe with its adjacent lateral occipital cortical regions. Among 16 prespecified speech motor control ROIs analyzed, cortical thinning in the ds-ALS group was observed in (1) bilateral middle motor cortices, (2) right posterior dorsal premotor cortex, and (3) left anterior cingulate cortex.

Our group-level whole-brain surface-based results revealed significant cortical thinning in left primary motor and somatosensory cortices in the ds-ALS group. This finding aligns with previously published *in vivo* neuroimaging and post-mortem studies in ALS (24, 25) and with those of Ferrea et al. (26), who found cortical thinning in the bulbar subregion of the precentral gyri in UMN-predominant ALS participants. The primary motor cortex is responsible for precise control and coordination of speech movements (27), and our results suggest a higher yield of focal cortical thinning in the somatotopically relevant facial-tongue-lip-laryngeal homuncular subregions of the sensorimotor cortex. Given that accurate and well-timed articulatory movements are critical for producing intelligible and fluent speech, cortical thinning in this region may contribute to well-documented deficits in intelligibility in speakers with ALS (28). While thinning in the motor cortex is an expected finding, deficits in the somatosensory cortex are less expected in the ALS population, as it has been long referred to as a “pure motor neuron disease.” Yet our finding of thinning in somatosensory cortex aligns with postmortem studies showing that in later stages of ALS, the pTDP43 burden spreads beyond the precentral gyrus to the postcentral gyrus and other neocortical regions including the cingulate gyrus (29–31). Furthermore, a growing amount of clinical evidence has supported the coexistence of sensory disorders in ALS (32, 33). This work highlights that the disease biology in ALS is multifocal and that the circuitries between the bulbar subregion of the primary motor and somatosensory cortices are anatomically and functionally connected.

In the right hemisphere, our group-level whole-brain surfaced-based results revealed thinning in inferior parietal lobe and lateral occipital lobe. Anatomically, there is no clear sulcal demarcation between the inferior parietal and the adjacent lateral occipital regions on the lateral cortical surface. It is, therefore, likely that there may be histological and functional overlaps between these two adjacent regions forming a composite region rather than interpreting these signals as arising from two functionally discrete regions. Previous studies investigating ALS have shown cortical thinning in the left homologs of these regions, which have been implicated in sensorimotor integration and identifying postures and gestures (26, 34, 35). Similarly, fMRI task activation studies have demonstrated the importance of bilateral inferior parietal lobe for integrating spoken and written language (36) and disambiguating speech signals (37).

Our *apriori* defined ROI analyses showed significant thinning in bilateral middle motor cortices in the ds-ALS group compared to HCs, which have been implicated in speech planning and initiation (27, 38). Difficulties in speech planning have been previously demonstrated in ALS, as prior work found significant more variability in phrase durations during reading compared to HCs (39).

Our ROI analyses also demonstrated cortical thinning in right posterior dorsal premotor cortex in the ds-ALS group. Of note, the premotor cortical area lies anterior to the primary motor cortex and its topographical organization is roughly the same as the primary motor cortex, with the facial-tongue-lips-laryngeal areas located on the dorsal/lateral aspect (40). The bulbar subregion of the premotor cortex has white matter connections with the superior longitudinal fasciculus and consistent connections with the pyramidal tracts (41), which are associated with more complex aspects of speech articulation (42) and motor planning of language function (43). Cortical thinning in these areas in the ds-ALS group may, therefore, underlie articulatory difficulties seen in this population, such as imprecise articulation and slow rate.

Our group-level ROI analyses lastly revealed cortical thinning in anterior cingulum gyrus in the ds-ALS group. Seminal work by Jürgens and Ploog (44) highlighted the role of anterior cingulum gyrus in the motivation to vocalize (or volitional speech initiation) in monkeys. More recent work conducted in humans similarly implicated the role of anterior cingulate cortex in modulating motor behavior during different affective states or levels of motivation (45). While the current study did not address motivation or affect in our participants, prior work has documented a lack of motivation to self-generate thoughts in individuals with ALS (46) and impairments in executive functions, emotion processing, and social cognition (47, 48).

Finally, our quantitative speech assessment in the six individuals with ALS included in our case analysis revealed that speaking rate was able to distinguish between those with and without cortical thinning. This finding is consistent with prior work reporting speaking rate as a key characteristic differentiating spastic dysarthria (relating to cortical/UMN deficits) from flaccid dysarthria (relating to LMN deficits) (49). While several speech characteristics, such as imprecision, are common in both spastic and flaccid dysarthria, slow speaking rate has consistently been associated with spastic dysarthria (49). Nevertheless, it is typically the *combination* of features that ultimately distinguishes between different dysarthria types. Indeed, in addition to imprecision, individuals with spastic dysarthria tend to present with strained voice and slow speaking rate (50). Similarly, individuals with flaccid dysarthria tend to also present with breathy voice and hypernasality (50). This result is consistent with prior work reporting speaking rate as a key characteristic differentiating spastic and flaccid dysarthria (49). Indeed, prior work posits that spastic dysarthria is related to cortical/UMN deficits, which contributes to speech characteristics such as strained voice and slow speaking rate (50). In contrast, flaccid dysarthria is related to LMN deficits, which contributes to speech characteristics such as imprecision, breathy voice, and hypernasality (50). Interestingly, our qualitative speech assessment found that two of the three individuals with cortical thinning (or potential UMN deficits) presented with spastic-dominant speech characteristics, while two of the three individuals with no cortical thinning (or potential LMN deficits) presented with flaccid-dominant speech characteristics. These results should be interpreted with caution, however, and they were based on a small case analysis.

Our study has limitations that are acknowledge here. The small sample size limits the generalizability of our findings. A larger sample size would provide more robust data and allow for more comprehensive statistical analyses, potentially revealing significant differences that our current study could not detect. For example, the small sample size might have contributed to our finding of no significant differences in cortical thinning between the ps-ALS group and HCs, as prior research has demonstrated such differences (51). However, we believe that the absence of thinning in the cortex in ps-ALS individuals is unclear and could be hypothesized to be multifactorial and may be explained by unanticipated sampling bias of participants showing either absence of UMN pathology and the presence of brainstem and spinal cord pathology only, or the microscopic reduction of Betz cells that is early and not easily visualized using current MRI morphology techniques. The authors acknowledge that using ALSFRS-R to define the ds-ALS and ps-ALS subgroups is a limitation of this study but that this is a preliminary data generating study. Future studies in a larger and longitudinal ALS cohort with deep phenotyping of speech, integrating more specific and sensitive tools for dysarthria assessment (e.g., voice signal analysis, artificial intelligence, precision medicine) would be logical expansion of this study as next steps (52). The small sample size introduces risk of unintended sampling bias and limits the generalizability of our findings. A larger and longitudinal sample would provide more robust data and allow for more comprehensive statistical analyses, potentially revealing significant differences that our current study could not detect. We did not conduct comparative analyses between ps-ALS and ds-ALS because of the small sample size and the pre-test risk of anatomical abnormalities in brain regions in both groups that could cancel some of the positive findings and confound the interpretation of results.

Overall, our findings provide evidence of the involvement of multiple cortical regions in speech deterioration in individuals with ALS, which is consistent with recent fMRI studies in healthy adults and clinical populations demonstrating the involvement of several neocortical pathways beyond the primary speech motor circuit system in speech production. A recent review by Riancho et al. (53) stated, “The elucidation of these ‘non-motor' systems involvement, which might also be part of the degeneration process, should prompt the scientific community to re-consider ALS as a pure motor neuron disease, which may in turn result in more holistic research approaches.” Genotypic information and other confounding variables, such as mood and cognition, should also be considered for all participants. Deep phenotyping of speech abnormalities in future longitudinal early and presymptomatic ALS cohorts will further our understanding of the multiregional spreading pattern of ALS and its association with speech deterioration.

## Data Availability

The raw data supporting the conclusions of this article will be made available by the authors, without undue reservation.
