# Supplementary material for: Speech motor impairment in ALS is associated with multiregional cortical thinning beyond primary motor cortex
Source: Front Neurol. 2024 Oct 1;15:1451177. doi: 10.3389/fneur.2024.1451177 (PMC11475245; doi:10.3389/fneur.2024.1451177)
Supplement: Supplementary file 1 [file Table_1.DOCX]

Supplementary Material

# Supplementary Tables

**Table 1.** Uncorrected and corrected p-values of the region of interest (ROI) analyses.

| ROI | ps-ALS vs. HC | | | | | | | | | | |  |
| --- | --- | --- | --- | --- | --- | --- | --- | --- | --- | --- | --- | --- |
|  | Right hemisphere | | | |  | | Left Hemisphere | | | |  |  |
|  | unadjusted p-value | | adjusted p-value | |  | | unadjusted p-value | | adjusted p-value | |  |  |
| aCG | 0.1 | | 0.65 | |  | | 0.13 | | 0.81 | |  |  |
| pCG | 0.58 | | 0.98 | |  | | 0.61 | | 1 | |  |  |
| aINS | 0.88 | | 0.98 | |  | | 0.85 | | 1 | |  |  |
| pINS | 0.54 | | 0.98 | |  | | 0.5 | | 1 | |  |  |
| SMA | 0.7 | | 0.98 | |  | | 0.51 | | 1 | |  |  |
| pre-SMA | 0.5 | | 0.98 | |  | | 0.65 | | 1 | |  |  |
| dIFo | 0.47 | | 0.98 | |  | | 0.7 | | 1 | |  |  |
| vIFo | 0.04 | | 0.4 | |  | | 0.71 | | 1 | |  |  |
| vPMC | 0.17 | | 0.77 | |  | | 0.95 | | 1 | |  |  |
| midPMC | 0.74 | | 0.98 | |  | | 0.92 | | 1 | |  |  |
| pdPMC | 0.11 | | 0.65 | |  | | 0.77 | | 1 | |  |  |
| mdPMC | 0.43 | | 0.96 | |  | | 0.57 | | 1 | |  |  |
| adPMC | 0.13 | | 0.67 | |  | | 0.14 | | 0.81 | |  |  |
| vMC | 0.99 | | 0.99 | |  | | 0.87 | | 1 | |  |  |
| dMC | 0.45 | | 0.97 | |  | | 0.4 | | 1 | |  |  |
| midMC | 0.21 | | 0.82 | |  | | 0.7 | | 1 | |  |  |
|  |  | |  | |  | |  | |  | |  |  |
| ROI | | ds-ALS vs HC | | | | | | | | | | |
|  |  | Right hemisphere | | | |  | | Left Hemisphere | | | | |
|  | | unadjusted p-value | | adjusted p-value | |  | | unadjusted p-value | | adjusted p-value | | |
| aCG | | **0.004** | | 0.07 | |  | | **0.0001** | | **0.01** | | |
| pCG | | **0.01** | | 0.14 | |  | | 0.12 | | 0.53 | | |
| aINS | | **0.03** | | 0.21 | |  | | 0.07 | | 0.42 | | |
| pINS | | 0.13 | | 0.4 | |  | | **0.02** | | 0.28 | | |
| SMA | | 0.15 | | 0.41 | |  | | 0.13 | | 0.53 | | |
| pre-SMA | | 0.28 | | 0.49 | |  | | 0.19 | | 0.56 | | |
| dIFo | | 0.45 | | 0.49 | |  | | **0.01** | | 0.2 | | |
| vIFo | | 0.07 | | 0.3 | |  | | 0.27 | | 0.62 | | |
| vPMC | | 0.22 | | 0.44 | |  | | 0.29 | | 0.62 | | |
| midPMC | | **0.006** | | 0.08 | |  | | **0.02** | | 0.29 | | |
| pdPMC | | **0.0006** | | **0.01** | |  | | **0.007** | | 0.15 | | |
| mdPMC | | 0.13 | | 0.4 | |  | | **0.013** | | 0.2 | | |
| adPMC | | 0.15 | | 0.41 | |  | | 0.56 | | 0.62 | | |
| vMC | | **0.02** | | 0.19 | |  | | **0.005** | | 0.1 | | |
| dMC | | **0.003** | | 0.07 | |  | | **0.01** | | 0.15 | | |
| midMC | | **0.00001** | | **0.01** | |  | | **0.0001** | | **0.01** | | |

**Table 2.** Darley, Aronson, and Brown Paradigm (1969a and 1969b).

| *Respiration (Breathing)* | *Phonation (Laryngeal)* | *Articulation* | *Resonance (Velopharyngeal)* | *Prosody* |
| --- | --- | --- | --- | --- |
| -Short phrases*  -Reduced loudness  -Monoloudness*  -Excessive loudness variation  -Loudness decay  -Forced expiration/ inspiration | -Low pitch*  -High pitch  -Monopitch* + **  -Pitch zbreaks  -Rough voice quality*  -Breathy voice quality**  -Strained voice quality *  -Diplophonia  -Vocal flutter  -Voice tremor  -Voice stoppages  -Audible inhalation/ inhalatory stridor  -Grunt at ends of phrases | -Imprecise consonants* + **  -Distorted vowels  -Irregular articulatory breakdown  -Articulatory blurring | -Hypernasality* + **  -Denasality or hyponasality (oral resonance on nasal consonants)  -Audible nasal emission/ nasal snort | -Slow rate*  -Fast rate  -Accelerating rate  -Variable rate  -Short rushes of speech  -Reduced stress*  -Excessive and equal stress  -Prolonged intervals  Inappropriate silences |

* = predominantly spastic symptom; ** = predominantly flaccid symptom
